# Sensory thresholds at different sites of the foot: a valuable reference for neurologic examinations

**DOI:** 10.1038/sc.2016.167

**Published:** 2016-11-29

**Authors:** J-W Zhang, Q Wang, X-F Wang, M-M Gao, X-P Yun, H-F Wu, Y Hong

**Affiliations:** 1 Department of Spine and Spinal Cord Surgery, China Rehabilitation Research Center, Beijing, China; 2 School of Rehabilitation Medicine, Capital Medical University, Beijing, China; 3 Department of Anesthesiology, China Rehabilitation Research Center, Beijing, China; 4 Department of Rehabilitation Medicine, Beijing Electric Power Hospital, Beijing, China

## Abstract

**Study design:**

Prospective healthy volunteer study for sensory thresholds.

**Objectives:**

The aim of this study was to test sensory thresholds at different sites of the foot to provide a reference for diagnosis and neurologic classification.

**Setting:**

A university hospital for the research and clinical practice of rehabilitation.

**Methods:**

Thirty healthy volunteers were recruited, and quantitative sensory testing was performed on three sites of the foot (medial malleolus (for the L4 dermatome), dorsum of the foot at the third metatarsal phalangeal joint (for the L5 dermatome) and lateral heel (for the S1 dermatome)). First, cold sense, warm sense, cold pain and hot pain were tested. Second, a monofilament tactility test was performed. Finally, a physical examination for sensation was performed.

**Results:**

All of the thresholds for the medial malleolus were significantly different from those for the dorsum of the foot at the third metatarsal phalangeal joint and lateral heel, whereas no significant difference existed between the values for the dorsum of the foot at the third metatarsal phalangeal joint and lateral heel.

**Conclusion:**

The sensory threshold of the human medial malleolus may be significantly different from those of adjacent sites of the foot. Thus, the results obtained from physical examination of sensory thresholds of the medial malleolus should be used modestly as a reference, but should not be used for diagnostic or classification purposes.

## Introduction

During a neurologic examination, dermatome refers to the area of the skin innervated by the sensory axons within each segmental nerve. A dermatome is often used to identify sensory deficits in clinical work. According to the International Standards for Neurological Classification of Spinal Cord Injury, which were revised in 2011 (ISNCSCI-2011),^1^ the sensory level is determined by performing an examination of the key sensory points, each presenting 1 of the 28 dermatomes of the spinal nerves. Appreciation of light touch and prick pin sensation at each key point is separately scored. Among these key points, the medial malleolus is designated as the L4 dermatome, the dorsum of the foot at the third metatarsal phalangeal joint is designated as the L5 dermatome and the lateral heel (calcaneus) is designated as the S1 dermatome. As ISNCSCI-2011 was used in our clinical work, the score of L4 was frequently lower than those of L5 and S1, even in patients in whom these nerves were spared. Does the medial malleolus show different sensitivity to sensory examination than other sites of the foot, even in normal subjects? To address this question, an investigation with quantitative sensory testing (QST) and a physical examination for sensation were performed on healthy volunteers, and the study results are reported here.

## Subjects and methods

**Figure 1:**
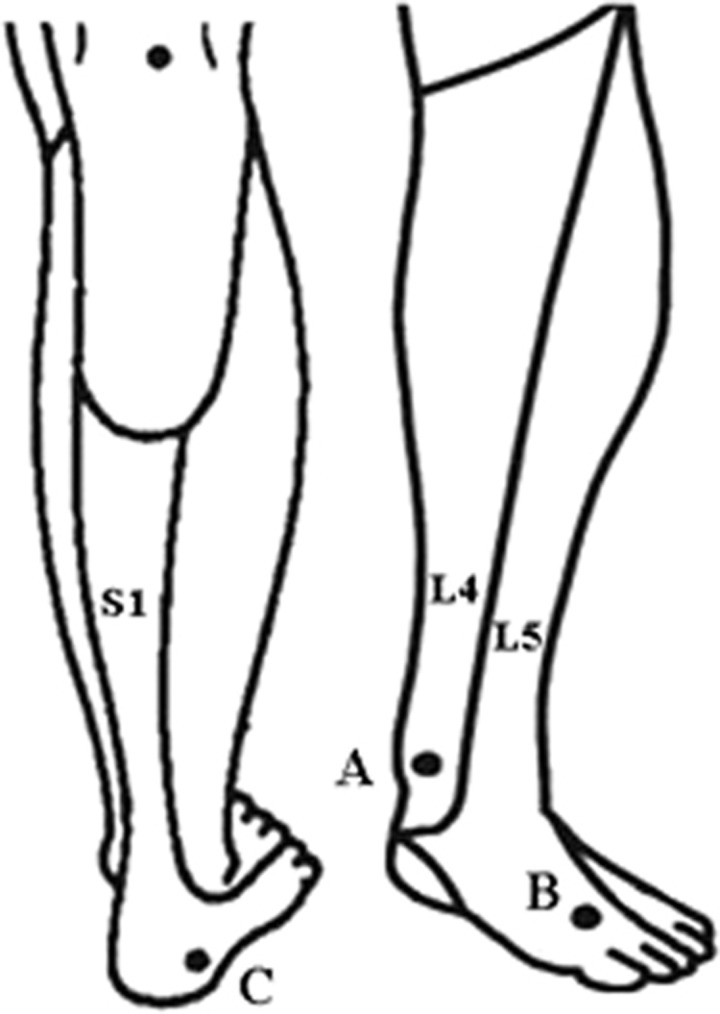
Key sensory points tested in the present study. (A) Medial malleolus, for the L4 dermatome. (B) Dorsum of the foot at the third metatarsal phalangeal joint, for the L5 dermatome. (C) Lateral heel (calcaneus), for the S1 dermatome.

Thirty healthy volunteers (10 males and 20 females), 20–60 years of age, were recruited. This study was approved by the ethical committee of China Rehabilitation Research Center, and written informed consent was obtained from each subject. None of the volunteers had diabetes or other diseases involving neurologic deficits. No medications were permitted during the testing period. The QST procedures were explained to the subjects by experienced physicians, emphasizing the importance of concentration and cooperation during testing. The temperature of the silent testing room was ~25 °C. The following sensory tests were performed at three sites of the foot, including the medial malleolus (referred to as A; for the L4 dermatome), the dorsum of the foot at the third metatarsal phalangeal joint (referred to as B; for the L5 dermatome) and the lateral heel (referred to as C; for the S1 dermatome; Figure 1). The tests were performed, and the test site did not exceed 1 cm from the center of site A, B or C. For each subject, three sites were tested bilaterally. Thus, two sets of data were obtained, and the differences between the right and left were ignored.

First, temperature sense was tested. Cold sense, warm sense, cold pain and warm pain were tested by the method of limits approach with a TSA-II tester (Medoc, Ramat Yishai, Israel) in all subjects. The equipment has a small probe with an area of 30 × 30 cm^2^ that can heat or cool the contacted skin by generating a temperature higher or lower than the skin. The temperature change was achieved through the current and a set of hot and cold water circulation devices. Usually, the default baseline of temperature was set at 32 °C, which would not result in a temperature sense with the probe contacting the skin for a few seconds. When measuring the threshold of the skin temperature sense, the temperature was changed at a rate of 1 °C s^−1^, with the probe temperature demonstrated on a monitor in real-time.

When testing temperature sense, the probe temperature was increased or decreased in a linear fashion. The subject was instructed to click a mouse button when he or she felt the sensation being tested. The temperature returned to the default value (32 °C) after each click. The probe temperature ranged between 0 and 50 °C. Warm and cold sense were tested 4 times with an interval of 5 s for each repetition. Warm and cold pain were tested three times with an interval of 10 s for each repetition. These intervals were used to ensure sufficient rest for the receptors of the skin. The calculated means were viewed as the quantitative thresholds of the skin temperature sense. The thresholds of temperature sense for A, B and C were compared and analyzed.

Second, monofilament tactility test was performed. Tactility of all subjects was performed by using Semmes-Weinstein monofilaments, 20 kinds of which are available with different specifications ranging from 1.65/0.0045 g to 6.65/447 g. The test was started using the monofilament with the smallest diameter contacting the skin perpendicularly, and the monofilament was slowly curved and maintained for 1.5. The subjects were asked whether they felt the monofilament make contact. If contact was not felt, a monofilament with a larger diameter was gradually used until the subject could feel the contact. The size of the monofilament that could be felt was recorded. The test of each monofilament was repeated three times at an interval of 1.5 to ensure a correct result. Finally, the pressure that elicited tactility was averaged and taken as the threshold of tactility.

Third, a physical examination was performed. Two aspects of sensation were examined for A, B and C according to ISNCSCI-2011.^1^ Appreciation of light touch and prick pin sensation at each site was separately scored on a 3-point scale, with comparison to the sensation on the patient's cheek as a normal frame of reference, as follows: 0=absent, 1=altered (impaired or partial appreciation, including hyperesthesia) and 2=normal or intact (similar to on the cheek). Light touch sensation was tested with a tapered wisp of cotton stroked once across an area that did not exceed 1 cm of skin with the eyes closed or vision blocked. Prick pin sensation (sharp/dull discrimination) was performed with a disposable safety pin that was stretched apart to allow testing on both ends; the pointed end was used to test for sharp sensation, and the rounded end of the pin was used to test for dull sensation. In testing for prick pin appreciation, the examiner determined whether the patient could correctly and reliably discriminate between sharp and dull sensations at each key sensory point. The inability to distinguish between dull and sharp sensations, as well as no feeling when being touched by the pin, was graded as 0. A grade of 1 was assigned when sharp/dull sensation was impaired. In this case, the patient reliably distinguished between the sharp and dull ends of the pin but stated that the intensity of sharpness may be greater or lesser than the feeling on the face. The key points were tested bilaterally for the L4, L5 and S1 dermatomes.

IBS SPSS Statistics, version 20.0, was used to perform statistical analyses in the present study. Data from the test of temperature sense and monofilament tactility test were expressed as the mean±s.d., and statistical analysis was conducted by one-way analysis of variance (ANOVA) followed by pairwise multiple comparison procedures using the least significant difference test and Student–Newman–Keuls test. Data from physical examination were taken as ranked data and were presented as median values (interquartile range) in the results. As the data sets did not have normal distributions, the statistical analysis was conducted by nonparametric testing using the Kruskal–Wallis and Mann–Whitney tests. The significance level was set at *P*<0.05.

## Results

The thresholds of the temperature senses of the 30 healthy subjects are shown in Table 1. The thresholds of warm sense and warm pain of A were significantly higher than those of B and C (*P*<0.05), whereas the thresholds of cold sense and cold pain of A were significantly lower than those of B and C (*P*<0.05). There was no significant difference between the thresholds of B and C (*P*>0.05). Tactility was elicited by 5 of the 30 kinds of Semmes-Weinstein monofilaments used in the present study (2.44/0.0275 g, 2.83/0.0677 g, 3.22/0.1660 g, 3.61/0.4082 g and 3.84/0.6958 g (number/pressure)). The thresholds of pressure at A, B and C are shown in Table 2. The threshold of pressure for tactility at A was significantly higher than those at B and C (*P*<0.05), whereas no significant difference existed between B and C (*P*>0.05). The sensory scores of A, B and C obtained according to ISCNSCI-2011 are shown in Table 3. The scores of A for light touch and pinprick appreciation were significantly lower than those of B and C (*P*<0.05), whereas the latter two showed no significant difference (*P*>0.05).

## Discussion

At the time of this study, ISNCSCI-2011 had been used for neurological evaluation of SCI cases in our institute since 2012. As we accumulated experience with it, we noted that the score for L4 was frequently lower than those for L5 and S1, even in patients with clear evidence supporting sparing of these nerves. Furthermore, in physical examination for lumbar degenerative disc disease, the sensory score for L4 was sometimes decreased if the medial malleolus was used as the checking point for L4, even if the motor function of L4 was normal. Does the medial malleolus show different sensitivity to sensory examination than other sites of the foot? To address this question, an investigation with QST and a physical examination for sensation at different sites of the foot innervated by L4 and adjacent dermatomes, L5 and S1, was performed.

Perceptual thresholds of different parts of the skin were studied by QST long ago not only for the evaluation of peripheral nervous system function in the clinical and research domains but also for the evaluation of spinal cord conduction.^2^ The temperature threshold obtained with a change rate of 3 °C s^−1^ was higher than that with a change rate of 1 °C s^−1^; thus a 1 °C s^−1^ change rate was used in the present study for more accurate results. In addition, gender had a negligible influence on the threshold,^3^ thus strict gender grouping was not adopted in this study.

According to this study, the perceptual thresholds of the medial malleolus were significantly higher than those of the dorsum of the foot at the third metatarsal phalangeal joint and lateral heel, as were the results of the light touch and pinprick tests (*P*<0.05, whereas the results of the latter two were nearly equal (*P*>0.05). The lateral painful and thermal stimuli were conducted through the spinothalamic tract to the brain, whereas tactile sense was conducted through the anterior spinothalamic tract.^4^ L4, L5 and S1 sensation was conducted through the corticospinal tract to the brain. For normal volunteers, it was considered unlikely that the difference in the perception thresholds of the three sites was caused by the spinal cord and brain. Therefore, the local anatomy and neurofibers were thought to have an important role in causing the difference. Cutaneous sensation consists of tactile, thermal and pain senses, the last of which incorporates pricking, thermal pain and visceralgia. Each sensation is spread from receptors to the spinal cord by specific nerve fibers.^5, 6^ The tests of sensation and the corresponding fibers were performed as shown in Tables 1,2,3 in the present study. Furthermore, the tests of monofilament tactile sense and light touch were performed using the same fibers, thus helping to demonstrate the objectivity of the light touch examination results. The responsible nerve fibers in the prick pin test were the same as those for cold sense and cold pain. Therefore, the results for cold sense and cold pain could act as corroborative evidence for the prick pin examination. Finally, a quantitative prick pin sensory examination may have been necessary for this study, but such an examination was beyond the scope of our laboratory.

On the basis of ISNCSCI-2011,^1^ key sensory points were assigned within each dermatome (enhanced with a prominence or fossa of bony and muscular anatomic landmarks) for expedient and correct use in clinical work. Light touch sensation is tested with a tapered wisp of cotton stroked once across an area not exceeding 1 cm of skin, which was the method used for testing in the present study. Furthermore, the results of the present study were consistent with what we have experienced in patients with spinal cord injuries; however, the purpose of this study was not to reveal loopholes in ISCNSCI-2011. In fact, it was hoped that the results of the present study would contribute to correct application of this standard.

In conclusion, sensation involving the human medial malleolus may be significantly different from that at adjacent sites of the foot. Thus, the results obtained from physical examination of sensation in the medial malleolus should be considered as a reference and should not be used for diagnostic or classification purposes. However, this conclusion was drawn only from our primary investigation with a short sample size because of the limit of the fund. Further research for the confirmation of the results has been planned, and it could be carried out as soon as the financial support was available.

## Data Archiving

There were no data to deposit.

## Figures and Tables

**Table 1:** Test of temperature sense

	*A*	*B*	*C*
Cold sense (°C)	26.5±2.2^a^	28.8±2.3	28.6±2.1
Warm sense (°C)	39.7±2.9^a^	36.1±2.4	36.4±2.2
Cold pain (°C)	13.4±5.7^a^	17.9±5.6	17±5.4
Hot pain (°C)	46.4±2.7^a^	44.1±2.2	44.3±2.6

a The thresholds of warm sense and warm pain of A are significantly higher than those of B and C (*P*<0.05), whereas those of B and C showed no significant difference (*P*>0.05).

**Table 2:** Monofilament tactility test

	*A*	*B*	*C*
Monofilament (g)	3.4±0.3^a^	2.8±0.3	2.8±0.4

a The threshold of pressure for tactility of A was significantly higher than those of B and C (*P*<0.05), whereas those of B and C showed no significant difference (*P*>0.05).

**Table 3:** Results of physical examination of sensation in the foot

	*A*	*B*	*C*
Light touch	1 (1,1)^a^	2 (2,2)	2 (1,2)
Pinprick	1 (1,2)^a^	2 (2,2)	2 (1,2)

^a^The scores of A for the light touch and pinprick tests were significantly lower than those of B and C (P<0.05), whereas those of B and C showed no significant difference (*P*>0.05).
